# Oregano Essential Oils Promote Rumen Digestive Ability by Modulating Epithelial Development and Microbiota Composition in Beef Cattle

**DOI:** 10.3389/fnut.2021.722557

**Published:** 2021-11-09

**Authors:** Rui Zhang, Jianping Wu, Yu Lei, Yunpeng Bai, Li Jia, Zemin Li, Ting Liu, Yangbin Xu, Jianxiang Sun, Ying Wang, Ke Zhang, Zhaomin Lei

**Affiliations:** ^1^College of Animal Science and Technology, Gansu Agricultural University, Lanzhou, China; ^2^Institute of Rural Development, Northwest Normal University, Lanzhou, China; ^3^Key Laboratory of Animal Genetics, Breeding and Reproduction of Shanxi Province, College of Animal Science and Technology, Northwest A&F University, Yangling, China

**Keywords:** oregano essential oil, rumen, microbiome, metabolome, VFAs, digestive enzyme, beef cattle

## Abstract

This study aimed to explore the effects of oregano essential oils (OEO) on the rumen digestive ability using multi-omics sequencing techniques. Twenty-seven castrated Pingliang red cattle were randomly separated into three groups (3 cattle/pen; *n* = 9) and fed on a daily basal diet supplemented with 0 (Con group), 130 mg (L group), and 260 mg (H group) OEO. The finishing trial lasted for 390 days, and all cattle were slaughtered to collect rumen tissue and content samples. We found that the rumen papillae length in the H group was higher than in the Con group. Amylase concentrations were decreased in the H group than the Con group, whereas the β-glucosidase and cellulase concentrations increased. Compared to the Con group, the relative abundance of propionate and butyrate in the H group was significantly higher. Higher relative abundance of *Parabacteroides distasonis* and *Bacteroides thetaiotaomicron* were observed with increasing OEO concentration. The function of rumen microbiota was enriched in the GH43_17 family, mainly encoding xylanase. Besides, metabolites, including heparin, pantetheine, sorbic acid, aspirin, and farnesene concentrations increased with increasing OEO dose. A positive correlation was observed between *Parabacteroides distasonis, Bacteroides thetaiotaomicron*, and β-glucosidase, cellulase and propionate. The abundance of *Parabacteroides distasonis* and *Parabacteroides_sp._CAG:409* were positively correlated with sorbic acid and farnesene. In summary, OEO supplementation increased the rumen digestive ability by modulating epithelial development and microbiota composition in beef cattle. This study provides a comprehensive insight into the OEO application as an alternative strategy to improve ruminant health production.

## Introduction

Residual antimicrobial agents caused by excessive use of growth-promoting antibiotics in feed pose an emergent threat to human health and the environment (1, 2). Banning the application of antibiotics as feed additives in livestock production has sparked great interest in exploring other natural pollution-free alternatives like plant secondary metabolites as substitutes for antibiotics and elucidating its mechanism of action (3). Oregano essential oils (OEO) is a potential alternative to growth-promoting feed antibiotics because of its broad-spectrum antimicrobial and anti-oxidant properties. Its main active ingredients include thymol, carvacrol, and terpinene (4). Different studies reported that they could inhibit the growth of pathogenic microorganisms by causing conformational changes in cell membranes to become less impermeable (5). Subsequently, growth-promoting effect (6), anti-diarrhea (7) and anti-oxidant function (8), microbial homeostasis regulation (9), etc., have been found to attract extensive attention of animal nutritionists.

Plant secondary metabolites, mainly essential oils, have been widely used in monogastric animals to provide nutritional regulation. They enhance growth phenotype (10), feed digestibility (11), and reduce diarrhea rate and oxidative stress (8, 12), particularly in piglets and broilers. However, the application of these materials in ruminants has been limited because of their complex digestive system. Previous studies reported that OEO has been associated with increases average daily gain (6) and feed utilization (13) in dairy cattle; and decreases diarrhea rate of neonatal calf (7, 14). As a fermentation site for forage and grain, the rumen is colonized by bacteria, protozoa, archaea and fungi, which play key roles in ensuring stability and increasing its ability to adapt to a wide range of dietary. Rumen epithelial cells absorbed nutrient substances into host internal circulatory system for energy supply. Therefore, the balance of rumen ecological environment and the development of rumen itself are critical for ruminant nutrition. Obviously, these microbes are closely interacted with the rumen pH value, fermentation parameters and epithelial development to further affect digestive ability. Evidences showed that OEO supplementation could regulate rumen fermentation parameters (15, 16) and microbiota composition in ruminants (17). Tea tree essential oils inhibit inflammatory cytokines expression in goat rumen epithelial cells (18). Our published data showed that supplementing OEO in goat feed improved growth performance and meat quality (19) and altered rumen fermentation parameters (20). Recently, the growth phenotype and meat quality of this study were described, indicating that OEO increased the average daily gain and feed conversion ratio (21). However, the phenomenon underlying these molecular system mechanisms remains largely unidentified. We speculated that diet supplementation with OEO would change rumen microbiota composition and function, influence the fermentation parameters and digestive enzyme activities and further promote epithelial development to increase rumen digestive ability. In this study, we used metagenomic sequencing and metabolomics to investigate the OEO effects on rumen microbiota composition and metabolic adaption of beef cattle. We aimed to provide new insights into the underlying mechanisms of OEO affecting the rumen digestive ability.

## Materials and Methods

This study was conducted at the Pingliang red cattle breeding center of Jingchuan County, Gansu, China. Animal sampling was approved by the Institutional Animal Care and Use Committee of the Gansu Agricultural University under permit NO.GAU-LC-2018-12.

### Animals, Diets, and Experimental Design

Twenty-seven castrated Pingliang red cattle, with an initial average body weight of 270.47 ± 16.26 kg (aged 12 months), were randomly assigned into three groups (3 cattle/pen; *n* = 9), and there were no statistically significant differences in initial body weight among the three groups (*P* > 0.05). The cattle were fed daily using the basal diet supplemented with 0 (Con group), 130 mg (L group), and 260 mg (H group) OEO. These OEO levels were obtained by adding its Rum-A-Fresh form (Ralco, Inc. Marshall, MN), containing 1.3% oregano oil and clinoptilolite as a feed-grade inert carrier. Beef cattle were fed a total mixed ration (TMR) consisting of corn silage and grain mixtures to meet or exceed their nutritional requirements outlined by the National Research Council (NRC 2003). For each group, the appropriate amount of OEO was accurately weighted and mixed with 1 kg grain daily and top-dressed to the feed bunk. The ration inclusion amounts were changed monthly (Supplementary Table S1), and all cattle were fed with fresh feed twice daily at 07:00 and 15:00. During the experimental period, all animals had access to feed and water *ad libitum*. Finally, the finishing plan lasted 390 days, after which all cattle were slaughtered.

### Sample Collection

At the end of the experimental period, all cattle were slaughtered after they fasted for 12 h. Subsequently, rumen tissue samples (about 2 × 2 cm) were immediately and carefully separated from the left dorsal sac to avoid squeezing and fixed in 4% paraformaldehyde solution for histological analyses using the hematoxylin-eosin (H&E) staining. Then, 5 mL of rumen content was collected from each animal, transferred into a sterile tube, immediately frozen using liquid nitrogen, and stored at −80°C awaiting DNA extraction. Another 15 mL rumen content was sampled and stored in a sterilized container at −20°C for digestive enzyme activities measurement and fermentation parameters evaluation. Rumen contents of all cattle were collected from the left dorsal sac in a mixture of liquid and solid components.

### Epithelial Parameters

Epithelial parameters, including rumen papillae length and width, were obtained through the H&E staining technique as previously described (22). Briefly, the fixed rumen tissues were dehydrated in ethanol, cleared in xylene, and embedded in paraffin blocks. Then, the cooling concretionary samples were sectioned at 5 μm thickness and mounted on glass slides. Paraffin sections were dewaxed to xylene (with water), rehydrated through graded ethanol series, rinsed in distilled water, and stained with H&E. Subsequently, the paraffin was sealed with neutral gum after being dehydrated again and immediately examined. Finally, a minimum of 10 well-oriented intact papillae length and width sections were photographed and measured using a light microscope (Motic BA 210, Xiamen, China) fitted with an image analyzer (Image-Pro Plus 6.0, Media Cybernetics, Bethesda, MD, USA).

### Digestive Enzyme Activities

The rumen content and homogenate medium mixture were placed in an ultrasonic pulp refiner to obtain a 10% homogenization buffer. Then, it was centrifuged at 2500 rpm at 4°C for 10 min, and the supernatant was subjected to β-glucosidase, cellulase, lipase, and amylase enzyme assays to determine concentration. Lastly, enzyme activities were measured using colorimetry method according to the instruction of reagent kits (Biosino Biotechnology Co. Ltd., Beijing, China; Mindray BS-240 automatic biochemical analyzer, Mindray, China).

### Fermentation Parameters

The pH value of the rumen content was immediately measured after the animal was slaughtered using Ark Technology PHS-10 portable acidity meter (Chengdu, China). The volatile fatty acids (VFAs) concentrations were tested as previously described (23). Briefly, the rumen content was centrifuged at 5,400 rpm for 10 min. We subsequently mixed 1 mL supernatant and a 0.2 mL 25% metaphosphate solution, containing 2 EB as an internal standard and uniformly mixed in a new centrifuge tube. After the reaction tube was immersed in an ice bath (30 min), it was centrifuged at 10,000 rpm for 10 min. The supernatant was passed through a 0.22 μm organic phase filter membrane and stored in 2 mL bottles awaiting subsequent analysis. A gas chromatograph (GC-2010, Agilent, Kyoto, Japan) fitted with an AT-FFAP capillary column (50 m × 0.32 mm × 0.25 μm) was used to determine the different VFAs concentrations. The column temperature was maintained at 60°C for 1 min, raised to 115°C at 5°C/min without reservation, and increased to 180°C at 15°C/min. Notably, the detector and injector temperatures were 260° and 250°C, respectively.

### DNA Extraction and Metagenomic Sequencing

Rumen content metagenomic DNA was extracted using the E.Z.N.A.^®^ Soil DNA Kit (Omega Bio-tek, Norcross, GA, U.S.) following the provided instructions by the manufacturer. Its concentration and purity were determined using TBS-380 and NanoDrop2000, respectively. Quality was checked on 1% agarose gel. Then, the DNA was fragmented to an average size of about 400 bp for paired-end library construction using Covaris M220 (Gene Company Limited, China). Consequently, paired-end sequencing was performed on an Illumina NovaSeq6000 platform (Illumina Inc., San Diego, CA, USA) at the Majorbio Bio-Pharm Technology Co., Ltd. (Shanghai, China). The paired-end Illumina reads were trimmed of adaptor sequences, and low-quality reads (length <50 bp or with a quality value <20 or having N bases) were removed using the fastp software (version 0.20.0) (24). Reads were aligned to the *Bos taurus* reference genome assembly using BWA (version 0.7.9a) (25), and any hit linked with the reads and their mated reads were discarded. Metagenomics sequence data were assembled using Multiple_Megahit (version 1.1.2) (26), contigs with lengths ≥300 bp were selected as the final assembling result. They were used for further gene prediction and annotation. The best candidate open reading frames (ORFs) were predicted using Metagene (27). The predicted ORFs with lengths ≥100 bp were retrieved and translated into amino acid sequences using the NCBI translation table. A non-redundant gene catalog cluster analysis was constructed using CD-HIT (version 4.6.1) (28) with 90% sequence identity and coverage. After quality control, all high-quality paired-end reads (with 95% identity) were mapped to the non-redundant gene catalog using SOAPaligner (version 2.21) (29), and the abundance of each gene in each metagenomics sample was evaluated. Subsequently, representative sequences of the non-redundant gene catalog were aligned to the NCBI NR database using BLASTP (Version 2.2.28+) (30) (with a best-hit e-value cutoff of 1e^−5^) to obtain annotation results and species abundance. Principal coordinates analysis (PCoA) was used to provide an overview of genetic differences among samples. The ORFs were aligned with the CAZy database using the hmmscan tool at an optimized e-value cutoff of 1e^−5^. Lastly, the Kyoto Encyclopedia of Genes and Genomes (KEGG) pathway annotation approach was performed against the KEGG database using a BLAST search (Version 2.2.28+) at an optimized standard cutoff e-value of 1e^−5^ (31).

### Metabolite Extraction and Metabolic Pathways Analysis

The rumen content samples were thawed on ice. Then, they were vortexed for 10 s. A total of 50 μL of rumen content sample and 150 μL precooled cold methanol (including 1 μg/mL 2-chlorobenzene alanine as internal standard) were vortexed for 3 min and centrifuged at 12,000 rpm at 4°C for 10 min. The supernatant was centrifuged at 12,000 rpm at 4°C for 5 min, and finally, the supernatant was collected into 2 mL bottles and used for subsequent LC-MS/MS analysis.

In the data acquisition system, we used Ultra-Performance Liquid Chromatography (UPLC, Shim-pack UFLC SHIMADZU CBM30A) and Tandem Mass Spectrometry (MS/MS) (QTRAP^®^). The UPLC running conditions were as follows: the chromatographic column used was Waters ACQUITY UPLC HSS T3 C18 (1.8 μm × 2.1 mm × 100 mm); column temperature was 40°C; flow rate was 0.4 mL/min; injection volume was 2 μL; the mobile phase consisted of eluent A (water, 0.1% formic acid) and eluent B (acetonitrile, 0.1% formic acid); and gradient elution was 95:5 V/V at 0 min, 10:90 V/V at 10.0 min, 10:90 V/V at 11.0 min, 95:5 V/V at 11.1 min, and 95:5 V/V at 14.0 min.

We acquired LIT and triple quadrupole (QQQ) scans on a triple quadrupole-linear ion trap mass spectrometer (QTRAP). Then, the QTRAP^®^ LC-MS/MS System, equipped with an ESI Turbo Ion-Spray interface, was operated in a positive and negative ion mode and controlled using the Analyst 1.6.3 software (Sciex). The following were the ESI source operation parameters: source temperature 500°C; ion spray voltage (IS) 5500 V (positive), −4500 V (negative); ion source gas I (GSI), gas II (GSII), and curtain gas (CUR) was set at 55, 60, and 25.0 psi, respectively; the collision-activated dissociation (CAD) parameter was set high. Instrument tuning and mass calibration were performed with 10 and 100 μmol/L polypropylene glycol solutions in QQQ and LIT modes, respectively. Finally, a specific set of MRM transitions were monitored for each period based on the metabolites eluted within that particular period.

### Statistical Analysis

All data were statistically analyzed using the One-way ANOVA procedure of SPSS (version 24.0). All data were presented as mean with SEM levels. GraphPad Prism version 8.0 software was used to conduct all analyses. Metagenomics statistics data were presented using the Kruskal-Wallis H test bar plots. Metabolome statistics data were analyzed based on retention time and ion current strength using the MultiQuant software to calculate the relative content of each compound. The Pearson's correlation coefficient analysis was conducted for the rumen microbiota, epithelial parameters, digestive enzyme activity, fermentation parameters, and metabolic profiles.

## Result

### OEO Increased Rumen Epithelial Parameters

**Figure 1:**
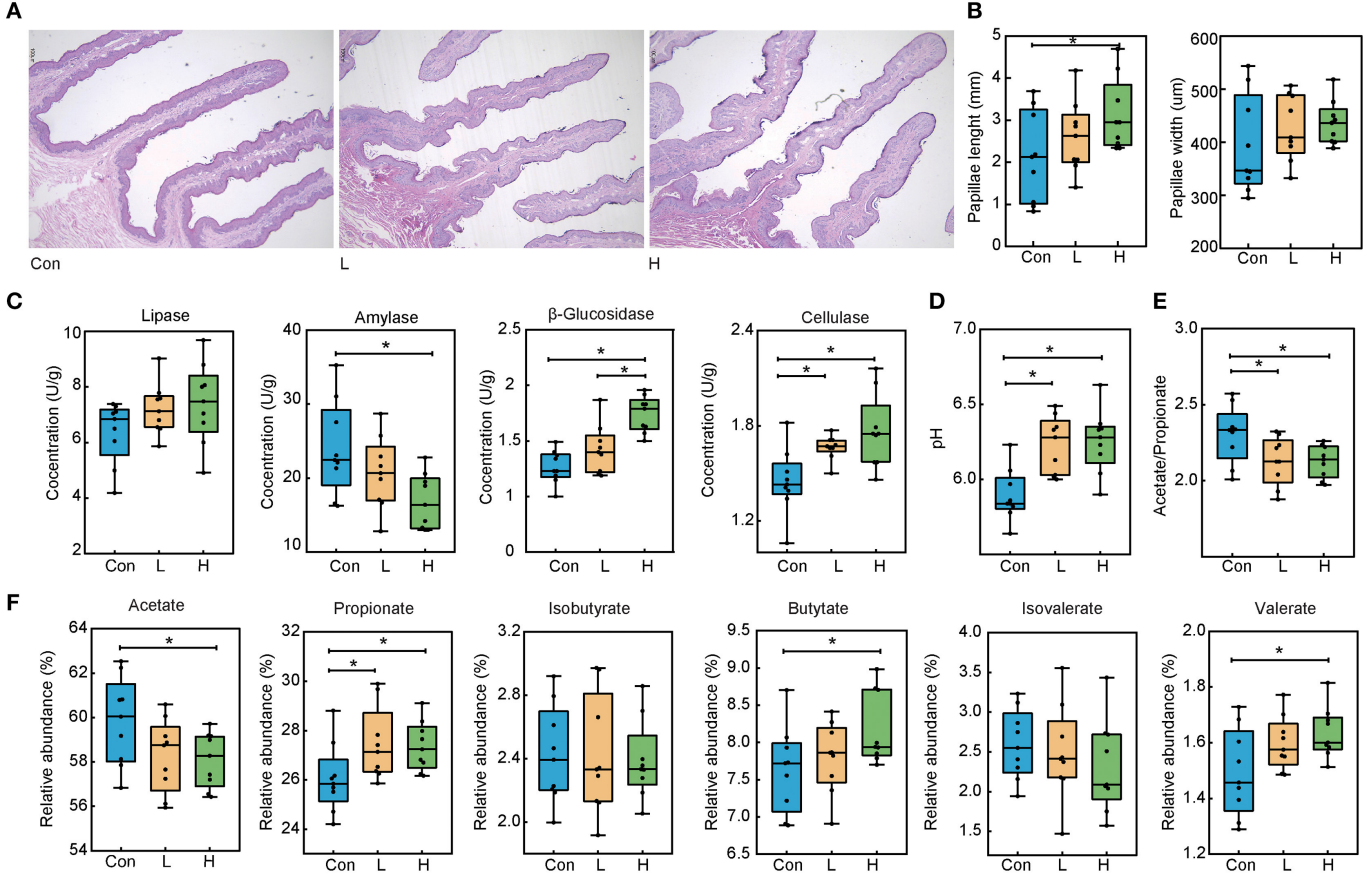
Effects of OEO on rumen epithelial parameters, digestive enzyme activities, and fermentation parameters in beef cattle. **(A)** Rumen papillae H&E staining result. **(B)** Increased rumen papillae length. **(C)** Increased rumen content β-glucosidase and cellulase concentrations and decreased amylase concentration. **(D)** Increased rumen content pH value. **(E)** Decreased rumen content acetate to propionate ratio. **(F)** Altered rumen content VFAs levels. *n* = 9 individuals/group. *P < 0.05.

Our previous study showed that dietary OEO supplementation strongly increased beef cattle average daily gain and feed conversion rate, this most likely to associated with promoting rumen digestive ability (Supplementary Figure S1). Here, we analyzed the papillae length and width via H&E staining to explore the effects of OEO on rumen epithelial parameters (Figure 1A). In the three experimental groups, papillae width was not significantly different (*P* > 0.05). On the other hand, papillae length in the H group was greater than in the Con group (Figure 1B, *P* < 0.05), which was increased by 31.68%. Therefore, OEO considerably improved rumen papillae length.

### OEO Changed Rumen Content Digestive Enzyme Activities

We measured the rumen content digestive enzyme activities to evaluate the rumen digestive ability (Figure 1C). Compared to the Con group, the OEO supplemented diet significantly decreased amylase concentration in the H group and increased its β-glucosidase compared to the Con and L groups (*P* < 0.05). Cellulase levels in the L and H groups were significantly increased than in the Con group (*P* = 0.001). Nevertheless, lipase levels were not significantly different in the three groups (*P* > 0.05).

### OEO Changed Rumen Fermentation Parameters

The pH and VFAs levels were determined to investigate the effects of the OEO supplementation on rumen fermentation parameters. The pH value was significantly increased in the L and H groups than in the Con group (Figure 1D, *P* < 0.001). Compared to the Con group, the relative abundance of propionate in the L and H groups was significantly increased (*P* = 0.043). The relative abundance of butyrate and valerate in the H group was higher than those in the Con group (*P* < 0.05), whereas the acetate levels were lower in the H group than in the Con group (*P* = 0.036). Notably, other VFAs were not significantly different in the three groups (Figure 1F, *P* > 0.05). Lastly, the lower acetate to propionate ratio was found in the L and H groups than in the Con group (Figure 1E, *P* < 0.05).

### OEO Changed the Composition and Functional Profiles of Rumen Microbiota

We further investigated the taxonomic and functional profiles of the rumen microbiota. Metagenomic sequencing of the microbial DNA extracted from 27 rumen content samples was performed. A total of 259.01 Gb high-quality reads were obtained, with an average of 9.59 Gb per sample. 2.51 million contigs with an average N50 length of 896.15 bp were assembled. A total of 33.90 million ORFs were predicted, and they had an average ORF length of 590.72 bp. Of note, the PCoA at the species level illustrated a slight difference in the three groups (ANOSIM, Bray-Curtis metric: *R* = 0.03, *P* = 0.20; Supplementary Figure S2). Briefly, the microbiota in the H group was closely clustered and slightly differentiated from those in the Con group. In contrast, the L group showed substantial cross fusion with the other two groups. Thus, compared to the Con group, OEO supplementation resulted in a considerable difference in the rumen microbiota of L and H groups.

**Figure 2:**
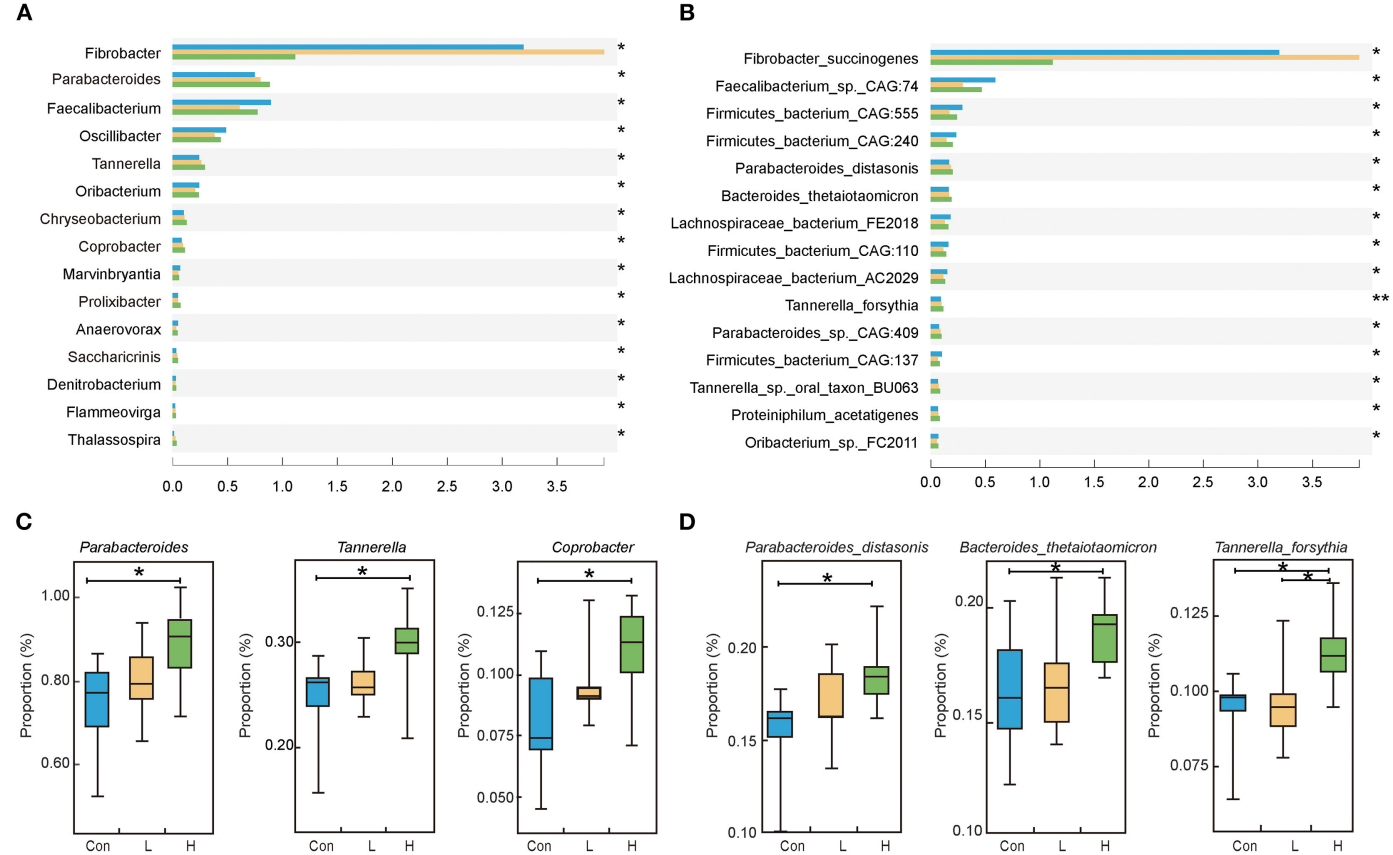
Rumen microbiota composition in beef cattle. **(A)** Differential bacteria at the genus level. **(B)** Differential bacteria at the species level. **(C)** The top three bacteria whose relative abundances significantly increased after OEO supplementation at the genus level. **(D)** The top three bacteria whose relative abundances significantly increased after OEO supplementation at the species level. *n* = 9 individuals/group. *P < 0.05, **P < 0.01.

We discovered that Bacteroidetes and Firmicutes are the predominant bacteria in beef cattle at the phylum level (*P* > 0.05, Supplementary Figure S3). We focused on screening microorganisms whose gradient varied with high OEO dosage based on the epithelial parameters, digestive enzyme, and fermentation parameters results. One hundred and fifty-seven different genera were identified in the three groups. Among them, *Parabacteroides, Tannerella* and *Coprobacter* had relatively high abundances and significantly increased with the addition of OEO (*P* < 0.05, Figures 2A,C). At the species level, 780 differential bacteria were identified among the three groups. However, the relative abundances of *Parabacteroides distasonis, Bacteroides thetaiotaomicron, Tannerella forsythia, Parabacteroides_sp._CAG:409*, and *Proteiniphilum acetatigenes* were considered the top five significantly increased after OEO supplementation (*P* < 0.05, Figures 2B,D).

**Figure 3:**
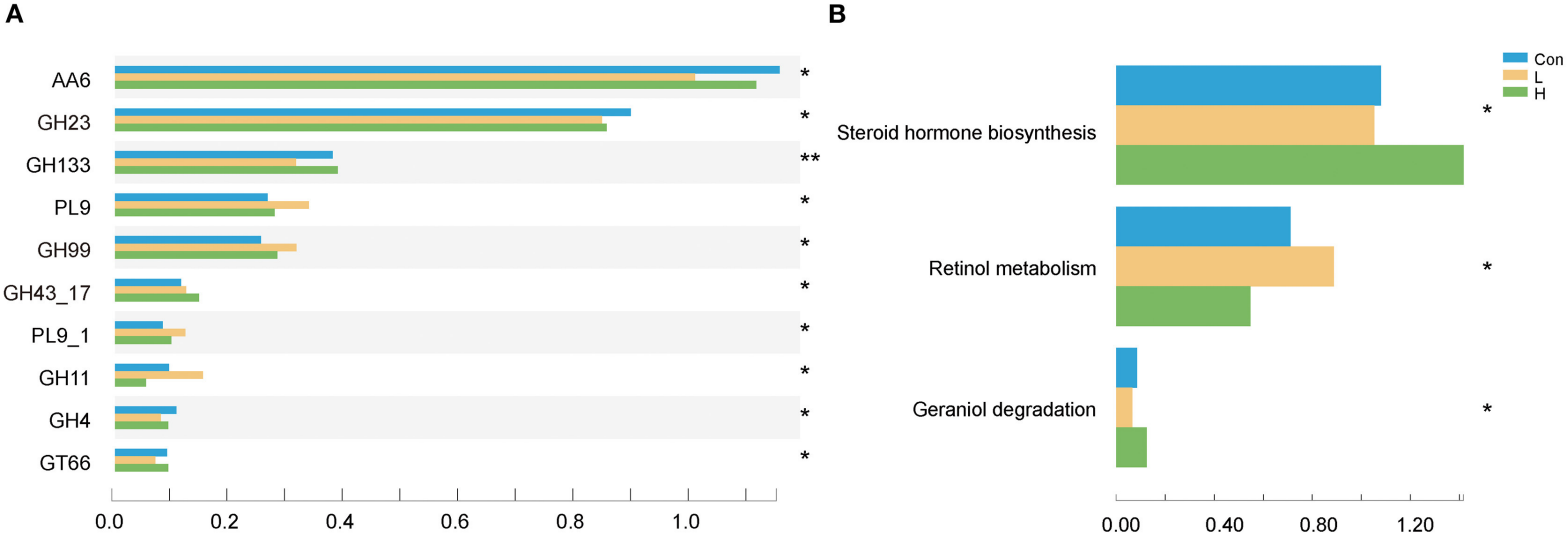
OEO diets changed rumen microbiota functional profiles. **(A)** CAZy genes with significant differences were selected. **(B)** Differential KEGG metabolic pathways were selected. *n* = 9 individuals/group. *P < 0.05, **P < 0.01.

We examined the CAZyme profiles of different lignocellulose degradation efficiencies. Here, the glycosyl hydrolase family of GH43_17 abundances were higher in the L and H groups than in the Con group (*P* < 0.05) and is usually involved in plant xylan degradation. Moreover, these genes corresponded to encode arabinanase (EC 3.2.1.99) and xylanase (EC 3.2.1.8). On the other hand, the GH23 family, which is involved in starch and glycogen degradation and encodes alpha-galactosidase (EC 3.2.1.22) and beta-galactosidase (EC 3.2.1.23), was considerably lower in the L and H groups than in the Con group (*P* < 0.05, Figure 3A). Furthermore, we determined that OEO expressed different KEGG functional potential. The genes involved in steroid hormone biosynthesis were highly enriched in the H group, while those in retinol metabolism were enriched in the L group (*P* < 0.05, Figure 3B).

### OEO Changed the Metabolite Profiles of Rumen

**Figure 4:**
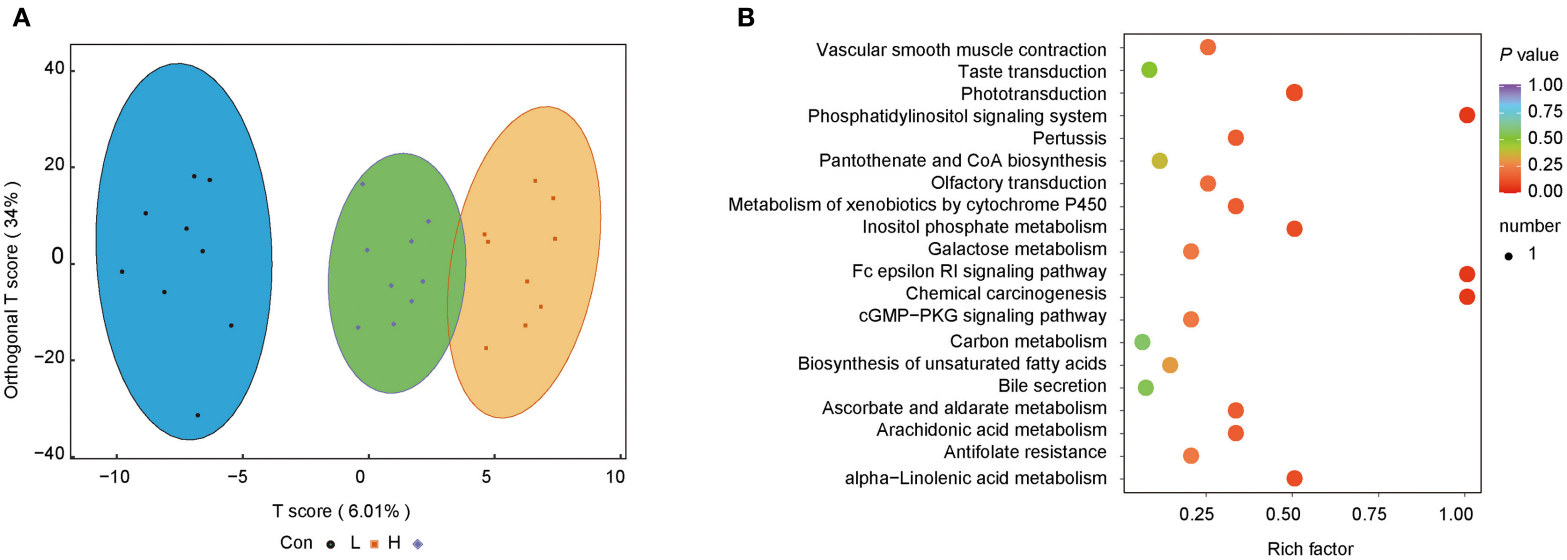
OEO diets changed rumen content metabolite levels in beef cattle. **(A)** OPLS-DA analysis. **(B)** Metabolic pathway enrichment analysis. *n* = 9 individuals/group.

We further investigated the difference in microbiota-induced metabolic profiles using LC-MS/MS. Five hundred and sixty metabolites were obtained from 27 rumen content samples. The OPLS-DA score plots showed that the three groups had a good separation of the rumen metabolites. The random permutation test indicated the satisfactory accuracy of the model (R^2^X = 0.452, R^2^Y = 0.942, *Q*^2^ = 0.31, Figure 4A).

The parameters of VIP > 1.0 and *P* < 0.05 were used as the criterion for separating the rumen metabolic compounds to assess which compounds were responsible for the differences in the three groups. Then, K-means clustering analysis was used to screen the relative abundance of metabolites gradient change with increasing OEO supplementation. Twenty-one differential compounds, including four aldehydes, ketones, esters (2-ethylhexyl phthalate, dibutyl phthalate, 9-octadecenal, and 2-pentyl-3-phenyl-2-propenal), four lipids [1-single palm essence, cis-11,14,17-eicosatrienoic acid, MAG (16:1) isomer, and MAG (16:1)], two organic acids (sorbic acid and aspirin), four alcohol and amines (2,2,2-trichloroethanol, methylamine, 2-nonanol, and heparin), two benzene (pyrene and 4-tert-butylbenzoic acid), one bile acid (taurocholic acid sodium salt hydrate), 1 Coenzyme and vitamins (pantetheine), and three others (alpha-cadinene, beta-cubebene, and farnesene) were obtained. Notably, these metabolites abundances were increased with increasing OEO supplementation (*P* < 0.05, Table 1).

**Table 1:** OEO diets changed rumen content metabolites in beef cattle.

**Number**	**Compounds**	**VIP**	***P***
1	2-Ethylhexyl phthalate	3.328	*P < 0.001*
2	Dibutyl phthalate	3.324	*P* < 0.001
3	Pantetheine	3.309	*P* < 0.001
4	Taurocholic acid sodium salt hydrate	2.999	*P* < 0.001
5	2,2,2-Trichloroethanol	2.950	*P* < 0.001
6	1-Single palm essence	2.949	*P* < 0.001
7	9-Octadecenal	2.699	*P* < 0.001
8	Cis-11,14,17-eicosatrienoic acid (C20:3)	2.667	*P* < 0.001
9	Sorbic acid	2.570	*P* < 0.001
10	MAG (16:1) isomer	2.555	*P* < 0.001
11	MAG (16:1)	2.555	*P* < 0.001
12	Alpha-cadinene	2.495	*P* < 0.001
13	Beta-cubebene	2.495	*P* < 0.001
14	Methylamine	2.421	0.004
15	Pyrene	2.329	0.004
16	2-nonanol	2.292	*P* < 0.001
17	2-Pentyl-3-phenyl-2-propenal	2.156	0.019
18	Farnesene	1.863	0.003
19	Heparin	1.753	0.049
20	Aspirin	1.530	0.038
21	4-tert-butylbenzoic acid	1.437	*P* < 0.001

Metabolic pathway enrichment analysis was conducted to identify the functional capacity of the different metabolic profiles. Herein, four metabolic pathways resulting from significantly different metabolites were obtained. Fc epsilon RI signaling pathway, inositol phosphate metabolism, phosphatidylinositol signaling system, and chemical carcinogenesis were highly enriched in the H group than the Con group (*P* < 0.05, Rich factor > 0.5, Figure 4B).

### Correlation Analysis Between Rumen Epithelial Parameters, Digestive Enzyme, Fermentation Parameters, Metabolite Profiles, and Microbiota

**Figure 5:**
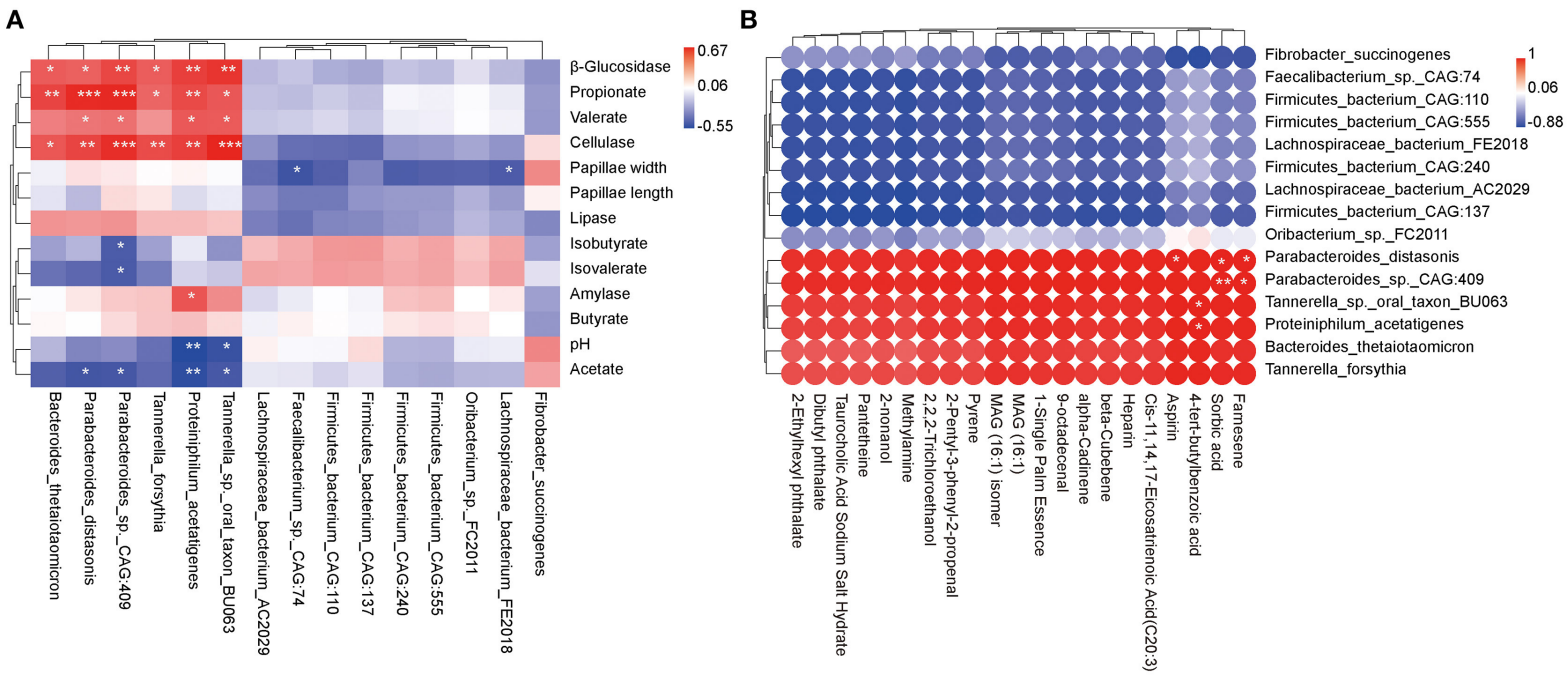
Correlation analysis between rumen epithelial parameters, digestive enzyme activities, fermentation parameters, metabolite profiles, and microbiota in beef cattle. **(A)** Pearman correlation heatmap differences between rumen epithelial parameters, digestive enzyme activities, fermentation parameters, and microbiota. **(B)** Pearman correlation heatmap differences between rumen metabolites and microbiota. *P < 0.05, **P < 0.01, and ***P < 0.001.

Species with significantly different abundances (*P* < 0.05) top 15 and rumen epithelial parameters, rumen digestive enzyme, and fermentation parameters were evaluated by regression analysis using the Pearson's correlation coefficient analysis (*R* > 0.4 or *R* < −0.4, *P* < 0.05; Figure 5A). The relative abundances of *Parabacteroides distasonis, Parabacteroides_sp._CAG:409, Proteiniphilum acetatigenes*, and *Tannerella_sp._oral_taxon_BU063* were positively correlated with β_glucosidase, cellulase, propionate, and valerate, whereas they were negatively correlated with acetate. Furthermore, the relative abundances of *Bacteroides thetaiotaomicron* and *Tannerella forsythia* were positively associated with β_glucosidase, cellulase, and propionate. Besides, amylase was positively linked with *Proteiniphilum acetatigenes*. Conversely, pH level was negatively correlated with *Proteiniphilum acetatigenes* and *Tannerella_sp._oral_taxon_BU063*; papillae width was negatively associated with *Lachnospiraceae_bacterium_AC2029* and *Faecalibacterium_sp._CAG:74*, and isobutyrate and isovalerate were negatively correlated with *Parabacteroides_sp._CAG:409*.

Subsequently, species with significantly different abundances top 15 and metabolites with VIP > 1.0 and *P* < 0.05 (Table 1) were analyzed using the Pearson's correlation coefficient (*R* > 0.4 or *R* < −0.4, *P* < 0.05; Figure 5B). The relative abundance of aspirin was positively correlated with *Parabacteroides distasonis*, whereas 4-tert-butylbenzoic acid was positively associated with *Proteiniphilum acetatigenes* and *Tannerella_sp._oral_taxon_BU063*. Finally, sorbic acid and farnesene were positively correlated with *Parabacteroides distasonis* and *Parabacteroides_sp._CAG:409*.

## Discussion

The growth performance, feed conversion rate, and carcass characteristics were previously published (21). Briefly, compared to Con group, the higher average daily gain and body weight were found in the L and H groups, which resulted in greater carcass weights. Moreover, OEO supplementation resulted in similar dry matter intakes among the three groups, but increased feed conversion rate by 9.33 and 16.04% for L and H groups. These data demonstrate that OEO diet improved the growth performance, feed efficiency, and carcass weight of cattle. However, the present study focused on rumen digestive ability, which is reported closely linked with epithelial development, microbiota composition, VFAs, digestive enzyme activities and metabolite profiles of rumen.

Rumen epithelial development is influenced by dietary intervention. A previous study reported that tylosin could promote papillae length in the dorsal sac of adult beef steers and reduce the abundance of *Fusobacterium necrophorum*. This prevents the inflammation of the rumen wall and enhances papillae development (32). Additionally, butyrate infusions stimulate cellular proliferation in the ruminal epithelial tissue of sheep (33). Interestingly, the present study established that OEO feed supplementation increases the papillae length of beef cattle, this may be correlated with high butyrate concentration in rumen. The change in papillae length could increase the nutrient contact surface area and further crush the feed to promote digestion and feed efficiency (34). Therefore, these findings demonstrated OEO is beneficial for rumen papillae development.

A digestive enzyme is a protein that promotes the hydrolysis of macromolecular materials into smaller molecules that can be easily absorbed by the body. Generally, digestive enzymes have substrate specificity characteristics; thus, complete digestion of complex structural polysaccharide plants requires specific enzyme combinations to break down diverse compounds (35). It has been reported that OEO increases fibrolytic bacteria abundance (i. e. *Fibrobacter succinogenes, Ruminococcus albus*, and *Ruminococcus flavefacien)* (36). Besides, it decreases amylolytic bacteria abundance because of reduced adhesion in readily digestible solids and starch (15, 37). Notably, these findings are similar to those observed in our study and indicate OEO could promote fiber digestive ability.

The pH value balance is an important index to evaluate the internal rumen environment. When the pH value is appropriate (6.0–6.3), rumen microbes could multiply and produce volatile fatty acid amounts to meet the host energy requirement. The rumen pH value is up-regulated after OEO supplementation, preventing rumen acidosis in current highly concentrated conditions. It is reported that the thyme essential oil (its main ingredient is thymol, 42.3% of DM) supplementation results in increased pH value in the rumen of cattle (38). This is because thymol inhibited the growth of lactic acid-producing bacteria (39). Furthermore, abrupt changes in rumen pH could affect microbiota and the fermentation products. This study has observed a high concentration in propionate, butyrate, and valerate and a low acetate concentration and acetate to propionate ratio. The lower acetate levels in the rumen denote less methane because methanogens could use acetate to produce methane (16). The high propionate concentration could attribute to energy supplementation via the gluconeogenic pathway to promote glucose synthesis (40). The low acetate to propionate ratio indicates a high energy efficiency in the feed (23). The butyrate would stimulate cellular proliferation of gastrointestinal epithelial tissue (17) and is recognized as an important mediator of gut microbiota regulation during energy homeostasis (41, 42). Study reported that EO feed increases propionate and butyrate metabolism in the intestinal microbiota (43). Moreover, the high nutrient levels and diet intake could reduce acetate concentration and increase the proportion of propionate (44). In the present study, OEO supplementation increased propionate and butyrate concentration. Several studies also obtained similar results in sheep after OEO supplementation (5, 9).

Like the changes observed in rumen fermentation parameters, the data on the taxonomic and functional annotation of the metagenome increased gene counts associated with energy metabolism after OEO supplementation. At the phylum level, OEO has no considerable effects on the relative abundance of Bacteroidetes and Firmicutes; however, at the genus level, the members of Bacteroidetes (*Parabacteroides, Tannerella*, and *Coprobacter genera*) are significantly increased following OEO supplementation. The primary Bacteroidetes function is to express several genes encoding carbohydrate-active enzymes, thus promoting the breakdown of the structural polysaccharide in the rumen to regulate glucose metabolism in the host animal (45, 46). This indicates that OEO could accelerate polysaccharide degradation to provide an adequate nutritional supply for rumen bacteria and the host. At the species level, *Parabacteroides distasonis*, which is the *Parabacteroides* mode species, could produce succinate to modulate host glucose metabolism (47). Succinate is a crucial intermediate toward microbial propionate synthesis (48). Simultaneously, it is an important precursor for the formation of glucose via the intestinal gluconeogenesis (IGN) pathway (40). Meanwhile, *Parabacteroides distasonis* increases the expression of the tight junction proteins and maintains the intestinal barrier integrity to promote digestibility and absorption capacity (49). The present study indicates that *Parabacteroides distasonis* positively correlated with propionate concentration. This could be because the former is a propionate-producing bacteria, as also reported by Wang et al. (47). *Bacteroides thetaiotaomicron*, an obligate anaerobe colonizing the gastrointestinal food particles and mucus, forms several polysaccharide utilization loci (PULs) to break down numerous dietary polysaccharides and provide genes to promote the acquisition and utilization of carbohydrates (45, 50). Evidence has shown that *Bacteroides thetaiotaomicron* could activate different genes to produce proteins attaching the polysaccharide-rich food surface and to degrade polysaccharides (51). Besides, *Bacteroides thetaiotaomicron* has been reported to confer keystone status during arabinogalactan degradation (52). Therefore, *Bacteroides thetaiotaomicron* has a higher ability to digest dietary fiber polysaccharides and host glycans. A positive correlation was found between *Bacteroides thetaiotaomicron* and β-glucosidase and cellulase levels. This could be due to the various glucoside hydrolase activities by the *Bacteroides thetaiotaomicron*., including β-glucosidase and cellulase activities (53). Thus, the increased abundance of *Bacteroides thetaiotaomicron* in the rumen after OEO supplementation might indicate the beneficial function of OEO in promoting rumen digestive ability. *Tannerella forsythia*, an obligate anaerobe colonized in the oral cavity, has been demonstrated as an essential periodontal pathogen (54). However, in this study, they have been considered as discriminative bacteria that respond to OEO utilization in beef cattle.

CAZyme degrade diet structural polysaccharides to provide nutrient substances for absorption by rumen epithelium. The diversity of carbohydrate compounds has been matched with specific enzymes that break down their bonds. For example, the GH43 family is primarily involved in encoding and degrading xylanase and xylan, respectively. Endo-1,4-xylanases (EC 3.2.1.8) targeted the backbone of xylan and converted it into short-chain xylose oligosaccharides and β-xylosidase (EC 3.2.1.37) could degrade xylose oligosaccharides to produce exlose. Then, α-L-arabinofuranosidase (EC 3.2.1.55) degrades side chains to acquire monosaccharides for the host (55). Apart from the GH23 family being involved in starch and glycogen degradation, it can also promote starch and glycogen transformation into dextrin to further break down the molecule into D-Glucose (56). Herein, the GH43 family genes were highly enriched in the H group. On the contrary, the GH23 family genes were highly enriched in the Con group, indicating OEO functional potential to degrade plant polysaccharides. These reports are similar to our data on the rumen digestive enzyme. Furthermore, KEGG functional taxonomy showed that retinol metabolism and steroid hormone biosynthesis were enriched in the L and H group; however, their abundance changes were not correlated with the increased in OEO dosage. Studies reported that retinol metabolism could maintain mucosal integrity and increase mucus production by the goblet cells (57); while the steroid hormone biosynthesis related with cholesterol production and regulated glucose and lipid metabolism. This illustrated that the function pathways of OEO was focused on energy metabolism.

Apart from affecting the rumen microbiota composition and function, OEO also alters the rumen metabolites and metabolic pathways. Some of these pathways are implicated in energy metabolism, cell growth, and immunoregulation. The Fc epsilon RI signaling pathway is initiated by the interaction between an antigen with immunoglobulin E (IgE) bound to the extracellular domain of the alpha chain of Fc epsilon RI. Subsequently, the activated mast cells release preformed granules, especially heparin, to increase the vascular permeability and further maintain rumen development (58). Conversely, pantothenate and CoA biosynthesis pathway plays a significant role in energy metabolism. Pantothenate is the precursor of CoA, and CoA is an essential enzyme in the TCA cycle. CoA also participates in the biosynthesis of fatty acids and cholesterol (59). Furthermore, about 85% of the dietary pantothenic acid is in CoA or phosphopentetheine forms, which is hydrolyzed by the ruminal microbiota into phosphopantetheine and pantetheine for energy provision. Previous studies showed that pantothenate could stimulate the ruminal cellulolytic bacteria (60) and that it might be correlated with *Flavobacteriia* abundance (61). Moreover, the abundance of heparin and pantetheine increased after dietary supplementation with OEO. Besides, A positive correlation was found between *Parabacteroides distasonis* and farnesene, sorbic acid, and aspirin. Farnesene belongs to the sesquiterpenoid family that has a similar action mechanism with monoterpenes. They are the main component in plants essential oils and have a broad spectrum in antimicrobial activity (62). It was reported that farnesene play a significant role during oxidative injury of eukaryotic cells (63). Sorbic acid, a food preservative and feed additive (64), could inhibit the dehydrogenase system of bacteria preventing their reproduction (65). Evidence shows that diets with sorbic acid exert prebiotic properties, stimulating the development of beneficial bacteria taxa (64). Aspirin, which has antibacterial and anti-inflammation activities, could prevent colorectal cancer by inhibiting cyclooxygenase enzymes and maintain intestinal barrier integrity (66, 67). Briefly, OEO supplementation alters rumen content metabolites and metabolic pathways. However, the further research is required to elucidate the causal relationship between microbiota and metabolites.

## Conclusion

In summary, OEO dietary intervention altered the rumen microbiota composition and function, increased rumen VFAs (propionate and butyrate) and enhanced the *Parabacteroides distasonis* and *Bacteroides thetaiotaomicron* abundance. Furthermore, the rumen digestive ability was increased by improving rumen epithelium papillae development, cellulase and β-glucosidase, heparin, pantetheine, farnesene, and sorbic acid concentration levels. Our study provided new insights into microbiota-mediated regulation of rumen digestive ability and provided a growth-enhancing dietary strategy involving the modulation of rumen microbiota.

## Data Availability Statement

The metagenomic data are available in the NCBI database under accession PRJNA730592.

## Ethics Statement

The animal study was reviewed and approved by the Institutional Animal Care and Use Committee of the Gansu Agricultural University under permit number No.GAU-LC-2018-12.

## Author Contributions

RZ, ZLe, and JW conceived and designed the experiments. RZ, YL, YB, LJ, ZLi, TL, YX, JS, and YW conducted the experiments and performed the statistical analysis of the experimental data. Finally, the paper was written by RZ and was modified by ZLe and KZ. All authors have read and approved the final manuscript.

## Funding

This research was financially supported by the Industry Support Project in Gansu Province (2020C-08), the Science and Technology Project of Gansu Province (17ZD2NC020), Modern Agriculture Industrial System Project of Gansu Province (GARS-CS-1), the Agricultural Special Project of Gansu Province (GSSLCSX-2020-1), and the Education Science and Technology Innovation Project of Gansu Province (GSSYLXM-02). The funding agencies did not participate in study design, data collection, analysis and interpretation or writing of the manuscript.

## Conflict of Interest

The authors declare that the research was conducted in the absence of any commercial or financial relationships that could be construed as a potential conflict of interest.

## Publisher's Note

All claims expressed in this article are solely those of the authors and do not necessarily represent those of their affiliated organizations, or those of the publisher, the editors and the reviewers. Any product that may be evaluated in this article, or claim that may be made by its manufacturer, is not guaranteed or endorsed by the publisher.

## Supplementary Material

Supplementary Figure S1 OEO increased average daily gain and feed conversion rate in beef cattle. **(A)** Increased average daily gain weight. **(B)** Increased feed conversion rate. *n* = 9 individuals/group. ^*^*P* < 0.05.

Supplementary Figure S2 PCoA of rumen content microbiota composition using ANOSIM of metagenomic sequences in beef cattle. *n* = 9 individuals/group.

Supplementary Figure S3 OEO changed rumen microbiota composition at the phylum level. *n* = 9 individuals/group.

Supplementary Table S1 Ingredient composition and nutrient levels of experimental diets for the respective feeding periods.
