# Supplementary figures and images for: Oregano Essential Oils Promote Rumen Digestive Ability by Modulating Epithelial Development and Microbiota Composition in Beef Cattle
Source: Front Nutr. 2021 Nov 9;8:722557. doi: 10.3389/fnut.2021.722557 (PMC8631176; doi:10.3389/fnut.2021.722557)

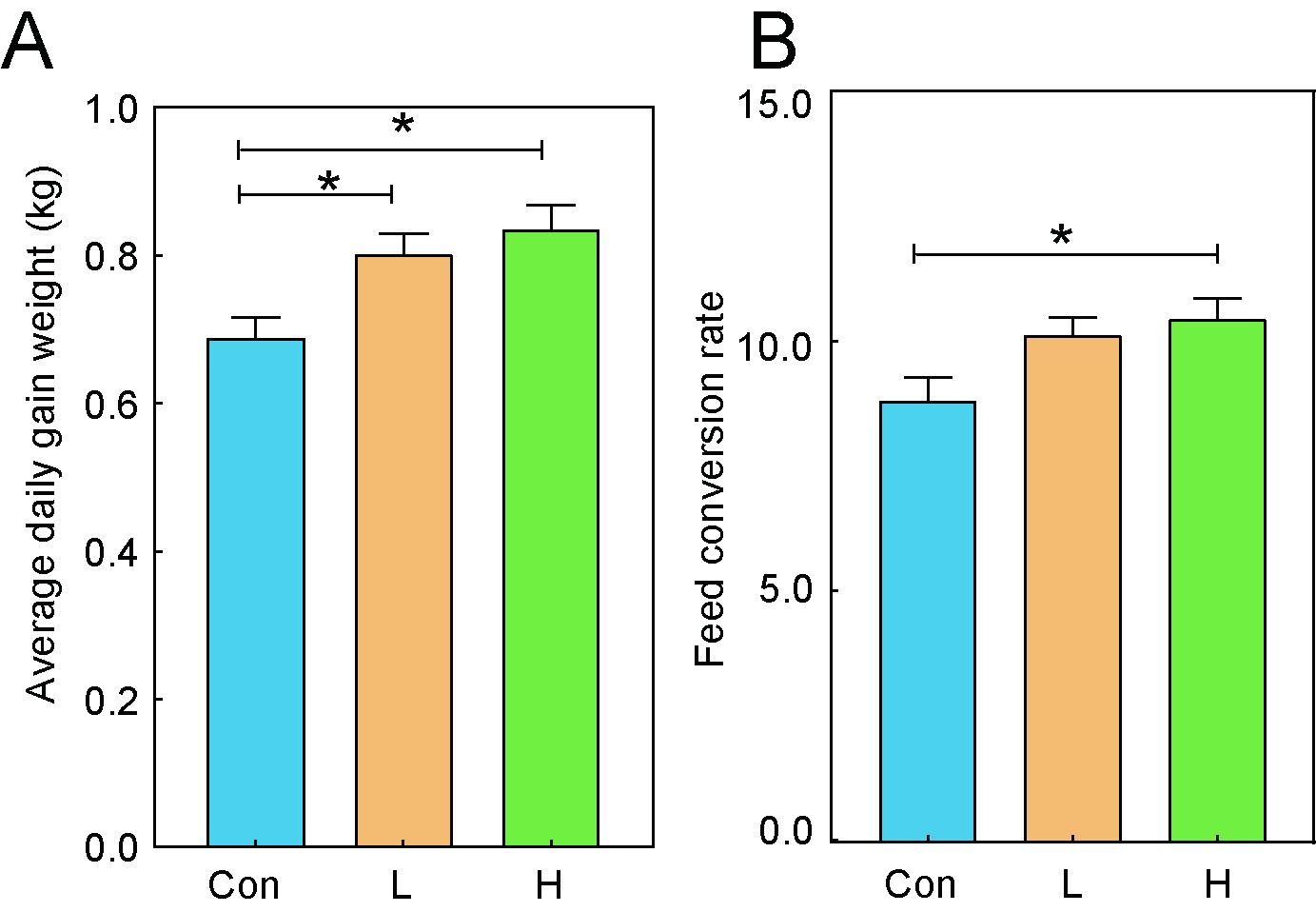

Supplement: Supplementary Figure S1 — OEO increased average daily gain and feed conversion rate in beef cattle. (A) Increased average daily gain weight. (B) Increased feed conversion rate. n = 9 individuals/group. *P < 0.05. [file Image_1.tif]

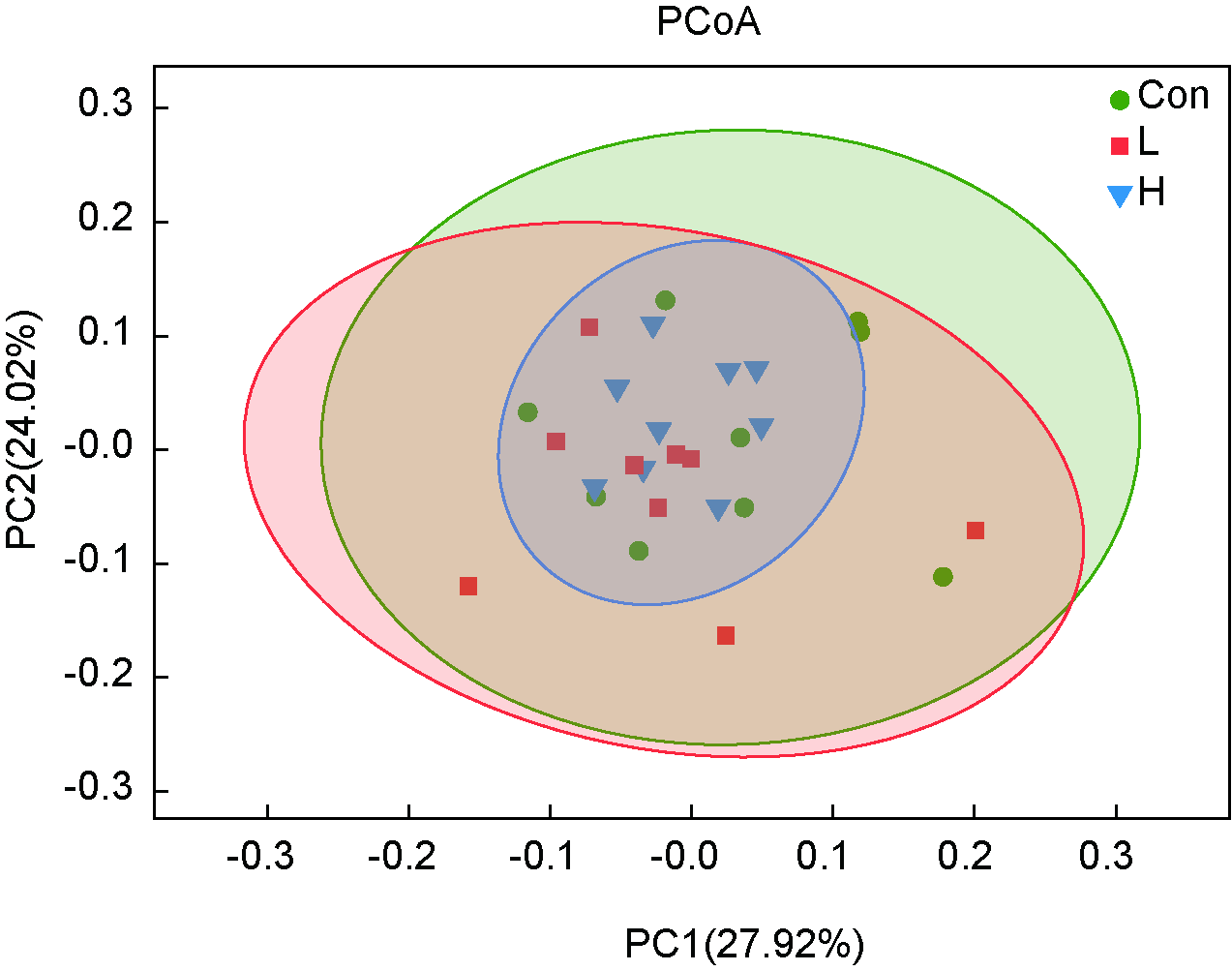

Supplement: Supplementary Figure S2 — PCoA of rumen content microbiota composition using ANOSIM of metagenomic sequences in beef cattle. n = 9 individuals/group. [file Image_2.tif]

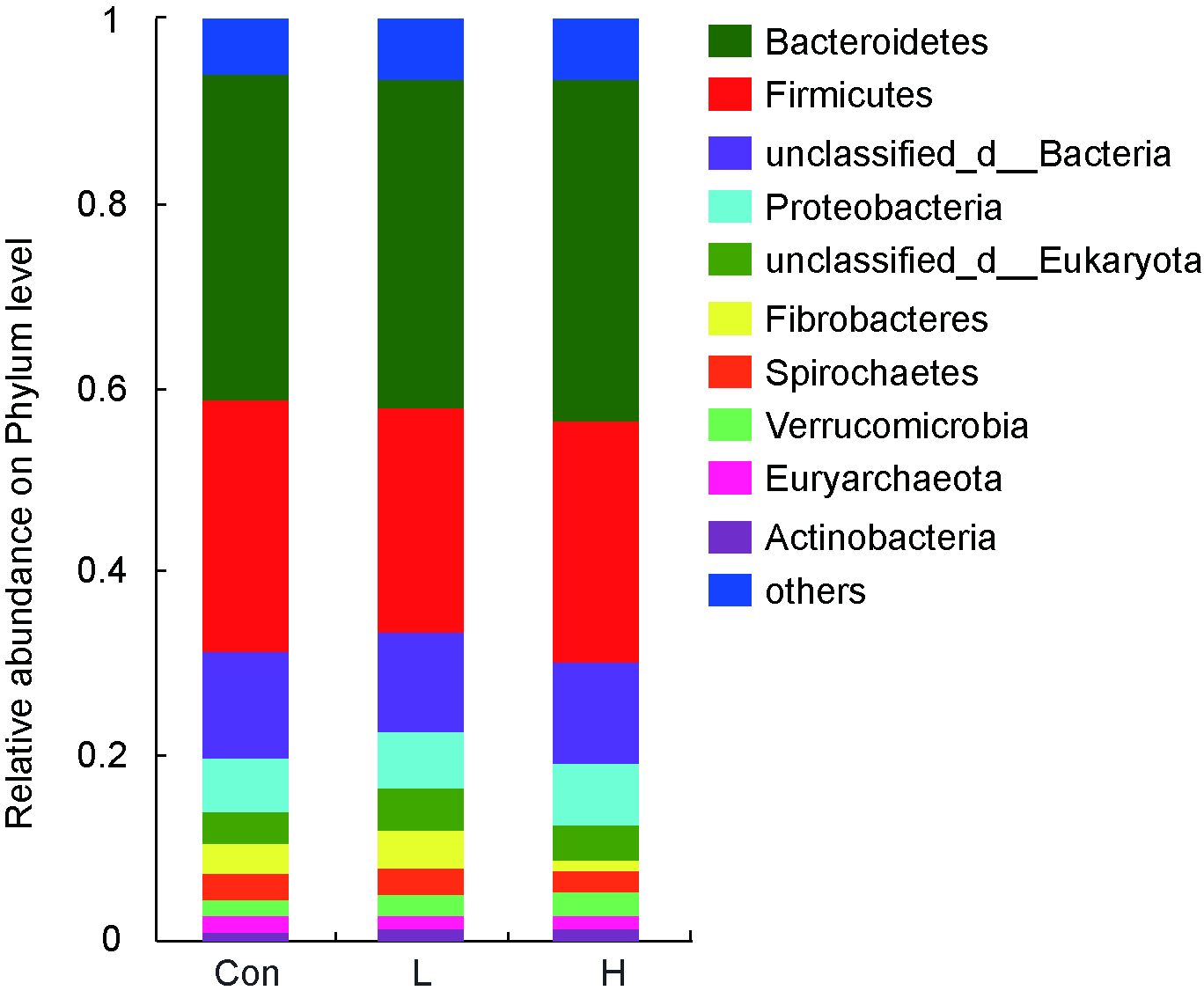

Supplement: Supplementary Figure S3 — OEO changed rumen microbiota composition at the phylum level. n = 9 individuals/group. [file Image_3.tif]
