# Supplementary material for: Oregano Essential Oils Promote Rumen Digestive Ability by Modulating Epithelial Development and Microbiota Composition in Beef Cattle
Source: Front Nutr. 2021 Nov 9;8:722557. doi: 10.3389/fnut.2021.722557 (PMC8631176; doi:10.3389/fnut.2021.722557)
Supplement: Supplementary Table S1 — Ingredient composition and nutrient levels of experimental diets for the respective feeding periods. [file Table_1.DOCX]

**Table S1.** Ingredient composition and nutrient levels of experimental diets for the respective feeding periods, % of DM

| Ingredients | Experimental period, month (day) | | | | | | | |
| --- | --- | --- | --- | --- | --- | --- | --- | --- |
|  | 1  30 d | 2  60 d | 3  90 d | 4~7  120~210 d | 8  240 d | 9  270 d | 10  300 d | 11~13  330~390 d |
| Steam-flaked corn | 21.85 | 34.36 | 30.89 | 40.00 | 56.00 | 61.00 | 61.00 | 65.00 |
| Steam-flaked wheat | 4.00 | 2.00 | 2.00 | 5.59 | 2.00 | 2.00 | 2.00 | 2.00 |
| Steam-flaked barley | 4.00 | 2.00 | 2.00 | 3.61 | 2.00 | 2.00 | 2.00 | 2.00 |
| Steam-flaked oatmeal | 477 | 8.24 | 2.87 | 8.00 | 9.84 | 2.00 | 4.72 | 4.72 |
| Corn germ meal | 4.82 | 2.00 | 8.33 | 2.00 | 3.01 | 8.72 | 6.00 | 2.00 |
| Corn DDGS | 16.00 | 6.14 | 10.00 | 3.19 | 3.00 | 3.00 | 3.00 | 3.00 |
| NaHCO_3_ | 0.67 | 0.64 | 0.67 | 0.75 | 0.92 | 0.94 | 0.94 | 0.94 |
| NaCl | 0.67 | 0.64 | 0.67 | 0.75 | 0.92 | 0.94 | 0.94 | 0.94 |
| Limestone | 0.90 | 0.70 | 0.60 | 0.63 | 0.50 | 0.50 | 0.50 | 0.50 |
| Premix^1)^ | 1.33 | 1.29 | 1.34 | 1.50 | 1.82 | 1.90 | 1.90 | 1.90 |
| Corn stalk silage | 41.00 | 42.00 | 40.62 | 34.00 | 20.00 | 17.00 | 17.00 | 17.00 |
| Total | 100.00 | 100.00 | 100.00 | 100.00 | 100.00 | 100.00 | 100.00 | 100.00 |
| Nutrient levels ^2)^ | | | | | | | | |
| CP | 12.60 | 11.55 | 11.27 | 11.30 | 11.95 | 11.46 | 10.09 | 10.09 |
| TDN | 72.15 | 72.26 | 72.00 | 73.99 | 77.35 | 78.07 | 77.87 | 77.87 |
| NEm（Mcal/100 kg） | 174.17 | 177.48 | 175.93 | 185.08 | 200.77 | 204.54 | 204.98 | 204.98 |
| NEg（Mcal/100 kg） | 112.33 | 114.71 | 113.45 | 122.65 | 136.44 | 139.95 | 140.39 | 140.39 |
| Ca | 0.40 | 0.40 | 0.35 | 0.36 | 0.28 | 0.27 | 0.27 | 0.27 |
| P | 0.39 | 0.32 | 0.38 | 0.31 | 0.32 | 0.31 | 0.29 | 0.29 |
| NDF | 29.31 | 26.20 | 28.38 | 22.63 | 17.51 | 16.03 | 15.87 | 15.87 |
| ADF | 16.02 | 15.38 | 15.67 | 12.90 | 8.92 | 7.95 | 7.87 | 7.87 |

^1)^ Contained (per kg): 900 IU of vitamin A, 150 IU of vitamin D, 25 IU of vitamin E, 10 mg of Cu, 80 mg of Fe, 20 mg of Mn, 40 mg of Zn, 1.0 mg of I, 0.6 mg Se.

^2)^ TDN、NEm and NEg were calculated values according to NRC (2003).
